# Determination of element composition and extraterrestrial material occurrence in moss and lichen samples from King George Island (Antarctica) using reactor neutron activation analysis and SEM microscopy

**DOI:** 10.1007/s11356-017-0431-2

**Published:** 2017-10-18

**Authors:** Tomasz Mróz, Katarzyna Szufa, Marina V. Frontasyeva, Vladimir Tselmovich, Tatiana Ostrovnaya, Andrzej Kornaś, Maria A. Olech, Jerzy W. Mietelski, Kamil Brudecki

**Affiliations:** 1 Institute of Biology, Pedagogical University of Cracow, Podchorążych 2, 30-084 Kraków, Poland; 2 Henryk Niewodniczański Institute of Nuclear Physics Polish Academy of Sciences, Radzikowskiego 152, 31-342 Kraków, Poland; 3 Joint Institute for Nuclear Rsearch, Joliot-Curie 6, Dubna, Russia 141980; 4 Borok Geophysical Observatory, A Branch of Shmidt’s Institute of Physics of the Earth of RAS, Borok, Nekouz, Yaroslavl region, Russia 152742; 5 Institute of Botany, Jagiellonian University, Kopernika 27, 31-501 Kraków, Poland

**Keywords:** Antarctica, Moss, Lichen, Biomonitoring, Space dust, Neutron activation analysis, SEM microscopy

## Abstract

Seven lichens (*Usnea antarctica* and *U. aurantiacoatra*) and nine moss samples (*Sanionia uncinata*) collected in King George Island were analyzed using instrumental neutron activation analysis, and concentration of major and trace elements was calculated. For some elements, the concentrations observed in moss samples were higher than corresponding values reported from other sites in the Antarctica, but in the lichens, these were in the same range of concentrations. Scanning electron microscopy (SEM) and statistical analysis showed large influence of volcanic-origin particles. Also, the interplanetary cosmic particles (ICP) were observed in investigated samples, as mosses and lichens are good collectors of ICP and micrometeorites.

**Electronic supplementary material:**

The online version of this article (10.1007/s11356-017-0431-2) contains supplementary material, which is available to authorized users.

## Introduction

**Fig. 1:**
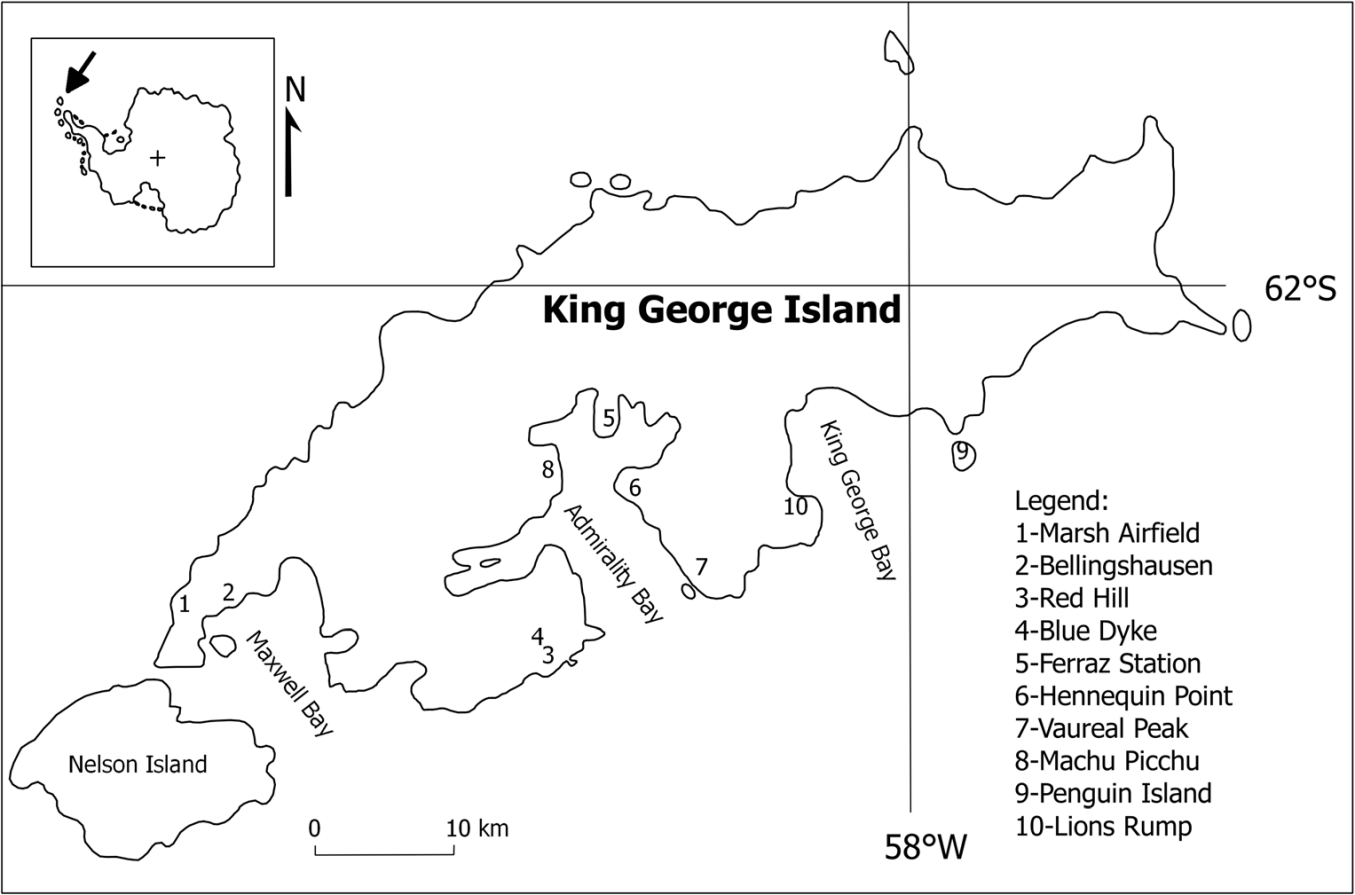
Map of King George Island with sampling sites (numbers)

The Antarctic region is still the least polluted area on our planet. It is isolated by ocean, cyclonic storm belts, and hard weather conditions (Shaw 1988). However, since the arrival of first explorers in the nineteenth century and increasing human activity (scientific activity and tourism) and threaten environment of this region (Osyczka et al. 2007; Chwedorzewska and Korczak 2010), as already reported, the main sources of pollution in Antarctic area are burning of fuels and waste storing (Kabata-Pendias 2000; Bargagli 2008). According to increasing human activity, it is necessary to monitor pollution concentration and to control this unique environment. The King George Island is an example of Antarctic area with high-human activity. Ten polar stations (eight all-years and two summers only) are located on the island (Osyczka et al. 2007). They belong to Argentina, Chile, Brazil, China, Ecuador, South Korea, Peru, Poland, Russia, Uruguay, and USA. The King George Island is one of the South Shetlands Island group (Fig. 1). The mean annual temperatures are in range from − 1.7 to 2.4 °C, and the ice-free area of the island is about 8% (Kejna 1999; Bӧtler 2011). Soils on King George Island are mainly derived from volcanic rocks like basalts and andesite basalts, but in few sites also sedimentary rocks are present, and the soils formed on these rocks are cryosols, leptosols, regosols, and fluvisols. In this environment, mosses and lichens are two of the most important groups of organisms. Mosses and lichens are protecting initial soils from weather conditions and thus forming basic environments, which can be later populated by microbes and lower organisms (Tatur and Myrcha 1984; Bӧtler 2011). Since 1970s mosses and lichens are used as indicators of environmental pollution, especially air pollution (Sloof 1993; Steiness 1995; Szczepaniak and Biziuk 2003; Wu et al. 2014). Mosses and lichens have no roots unlike higher plants. Therefore, their basic nutrient source is atmospheric elements deposition. Because these groups of organisms have no protective cuticle, they can absorb ions from the air, rainwater, or snow. The growth rate of mosses and lichens is slow, so they can accumulate pollutants very effectively (Nash 1996; Turetsky et al. 2012). In this study, lichens *Usnea antarctica* (Du Rietz), *U. aurantiaco-atra* (Jacq.), and moss *Sanionia uncinata* (Hedw.) were used as bioindicators of concentration of heavy metals, rare earth elements, and trace and major elements in the air. Analysis of samples was carried out using Instrumental Neutron Activation Analysis (INAA). INAA is a non-destructive, multi-elemental method that allows simultaneous determination of about 50 elements in a single sample with mass about 300 mg (Bode 1996; Frontasyeva 2011). The advantages of INAA are low-detection limits, very precise results, and fast-sample preparation without complicated chemical treatment, thus INAA is widely applied in environmental pollution studies, for example, in the Intercontinental Cooperative Programme on Effects of Air Pollution on Natural Vegetation and Crops (ICP Vegetation) research (Kłos et al. 2013; Thinova et al. 2014; Allajbeu et al. 2016; Harmens et al. 2016). The goal of this study was to estimate local environmental pollution levels and identify possible pollution sources including not only anthropogenic but also natural like, e.g., volcanic or biogenic activity, and analyze differences between trace element accumulation by mosses and lichens including investigation of rare earth elements distribution. Additionally, scanning electron microscopy (SEM) was applied to determine microparticles and presence of cosmic dust in moss and lichen samples. Cosmic dust is very abundant on earth (Brownlee 1985). The total mass of dust inside earth orbit is about 10^15^ tons, and every year 4∙10^3^–10^4^ tons is deposited on earth surface (Yada et al. 2004; Grachev et al. 2008) that means that cosmic dust deposition should be taken account during environmental trace analysis (Yada et al. 2004). Investigations of cosmic dust were started by the HMS *Challenger* expedition, during which traces of cosmic matter were discovered in sea sediments (Murray and Renard 1891). Later, metallic microspherules have been found also in Antarctic and Greenland glaciers and on deserts (Maurette et al. 1986; Yada et al. 2004). Cosmic dust can be subdivided into interplanetary cosmic dust particles (IDP) or micrometeorites depending to size of particles (IDP < 30 μm and micrometeorites > 50 μm), but there are no existing rigorous criteria for discrimination between cosmic dust and micrometeorites yet (Genge et al. 1997; Grachev et al. 2008). Cosmic dust particles may have very different shapes, so not only morphological criteria (spherical shape, textures, or metallic luster) but also their chemical composition is very important for confirmation of their cosmic origin. For example, volcanic-origin Fe microspheres have high-Ti content (more than 10%) and rarely have perfect spherical shape (Szӧőr et al. 2001; Grachev et al. 2008). There is still no clear origin of cosmic dust. It can be remained by primordial matter from protoplanetary cloud, or it can be produced by comets and asteroids destruction (Grachev et al. 2008).

## Materials and methods

Moss and lichen samples were collected in summer seasons 2002–2003 by Polish Antarctic Expeditions of the Polish Academy of Sciences to the Henryk Arctowski Station. Sampling sites were free from direct influence of visible aerosols. Four sampling sites (1, 2, 5, and 8) were located close to polar stations. Sampling site 1 was located near Marsch Airfield, close to Chilean, Eduardo Frei base with average winter population of 80 people. Samples from sampling site number 2 were collected close to Russian Bellingshausen base with average winter population of 25 people. Sampling site number 5 was located near Brazilian station Comandante Ferraz with 12 people winter crew, and site number 8 was located close to Peruvian Macchu Picchu station (summer only station). *S. uncinata* (Hedw.) is one of the most common mosses in the Antarctic region (Putzke et al. 2015). It forms large area carpets, associated with other mosses and lichens. *U. antarctica* (Du Rietz) and *U. aurantiacoatra* (Jacq.) are lichens mostly associated with *S. uncinata* (Victoria et al. 2009; Victoria et al. 2013). Sampling sites (Table 1) were located within Antarctic stations operating in full-year or summer only cycle. Collected material was packed into plastic bags. After identification, samples were dried and shipped to Poland. In 2015, samples were analyzed using INAA in Frank Laboratory of Neutron Physics, Joint Institute for Nuclear Research in Dubna. Samples were once again dried in 30 °C and cleaned mechanically from possible soil impurities using scissors and tweezers. From each sample, two portions about 300 mg were taken to form two tablets for short and long irradiation. The mass of 300 mg is a sufficient mass to use moss and lichen samples without homogenization (Korzewka et al. 2007; Steinnes et al. 2007). Tablets were packed in polyethylene bags and plastic containers (for short irradiation) and in aluminum foil and aluminum container for long irradiation. Samples were irradiated in IBR-2M reactor in the Frank Laboratory of Neutron Physics. For determination using short-lived isotopes (Mg, Al, Cl, Ca, V, Mn, I, Cu, K, S, Ti, In, Dy, Si), samples were irradiated for 3 min. After short irradiation in channel 2 (without Cd screen, Table 2), samples were measured twice. First measurement was for 5 min after 3 min of decay and second one for 20 min after 10 min of decay. For determination of long-lived isotopes (Na, K, Sc, Cr, Fe, Co, Ni, Sb, Zn, As, Rb, Sr, Cs, Ba, La, Sm, W, Th, U, Cs, Au, Eu, Nd, Zr, Rb, Br, Se, Cd, Ag, Mo, Ce, Hg, W, Ta, Hf, Lu, Yb, Tm, Tb, Gd), samples were irradiated by 100 h in Cd-screened channel 1. After irradiation, samples were repacked and measured for 45 min and for 3 h after 5 and 20 days, respectively. Measurements of gamma rays were performed using High-Purity Germanium (HPGe) detector with resolution 1.9 keV on 1332-keV line from ^60^Co. Data acquisition process was controlled using Genie 2000 software (Canberra). The processing of data and determination of element concentration in samples were performed using software developed in FLNP JINR (Ostrovnaya et al. 1993; Ostrovnaya 2000). For quality assurance purposes, certified reference materials (IAEA 336) and neutron flux comparators were used.

**Table 1:** Sample description

No.	Species	Location	Coordinates	Sample code
Lichens				
1	*Usnea antarctica*	Marsh Airfield	62°11′39,5″ S	i-01
			58° 58′35,0″ W	
2	*Usnea antarctica*	Red Hill	62°13′59,98″ S	i-02
			58°30′00″ W	
3	*Usnea antarctica*	Ferraz Station	62°05′00″ S	i-03
			58°23′28″ W	
4	*Usnea aurantiaco-atra*	Bellingshausen	62°12′ S	i-04
			58°58′ W	
5	*Usnea aurantiaco-atra*	Hennequin Point	62°07′13,0″ S	i-05
			58°23′46,4″ W	
6	*Usnea antarctica*	Penguin Island	62°06′15,6″ S	i-06
			57°59′40,2″ W	
7	*Usnea antarctica*	Vaureal Peak	62°10′58,2″ S	i-07
			58°17′32,3″ W	
Mosses				
1	*Sanonia uncinata*	Hennequin Point	62°07′13,0″ S	j-01
			58°23′46.4″ W	
2	*Sanonia uncinata*	Marsh Airfield	62°11′18,5″ S	j-02
			58°59′56.2″ W	
3	*Sanonia uncinata*	Bellingshausen	62°12′ S	j-03
			58°58′ W	
4	*Sanonia uncinata*	Machu Picchu	62°05′30″ S	j-04
			58°28′14″ W	
5	*Sanonia uncinata*	Blue Dyke	62°13′30″ S	j-05
			58°28′14″ W	
6	*Sanonia uncinata*	Vaureal Peak	62°10′52,8″ S	j-06
			58°17′32,3″ W	
7	*Sanonia uncinata*	Ferraz Station	62°08′65″ S	j-07
			58°39′32″ W	
8	*Sanonia uncinata*	Penguin Island	62°06′ S	j-09
			57°56′ W	
9	*Sanonia uncinata*	Lions Rump	62°08′01″ S	j-10
			58°07′25″ W

**Table 2:** Neutron flux parameters of irradiation channels

Irradiation channel	Ф∙10^12^ [*n*∙cm^−2^∙s^−1^]	Ф∙10^12^ [*n*∙cm^−2^∙s^−1^]	Ф∙10^12^ [*n*∙cm^−2^∙s^−1^]	*T* (°C)
	0 *< E <* 0.55 [eV]	0.55 *< E* < 10^5^ [eV]	10^5^ < *E* < 25∙10^6^ [eV]	
	Thermal	Resonance	Fast	
Ch1 (Cd-screened)	0.02	3.3	4.2	70
Ch2	1.2	3.0	4.1	60

SEM microscopy and MPA analysis were performed using Tescan Vega II (Tescan, Czech Republic) with energy dispersive X-ray analyzer (EDS). Samples were analyzed with an accelerating voltage of 20 kV and beam current 0.2 nA. Samples of mosses and lichens with mass about 300 mg were milled in the agate mortar and sonicated. After homogenization, magnetic particles were extracted by permanent magnet. Magnetic particles collected on the magnet were transferred to double layer conductive carbon adhesive tape and placed on objective table using glass rod. Data acquisition time was 1–2 min, and obtained results were normalized to 100% (Pechersky et al. 2015a, b). Total number of 53 objects found in 14 samples of mosses and lichens were analyzed. Depending on size of object, 3 to 10 points were analyzed by EDS spectrometer.

Principal component analysis (PCA) was used for identification possible sources of elements in analyzed samples. For PCA analysis, we used Varimax rotation with Kaiser normalization (eigenvalues > 1) and *p* = 0.05.

Normalization of REE concentrations was done by using chondrite values following by Taylor and McLennan (1985).

## Results and discussion

Tables 3 and 4 show ranges and medians for 52 element concentrations determined in moss and lichen samples. The concentrations of elements in investigated samples were different depending on sampling site and species. In general, concentrations of elements in moss samples were greater than in lichens. This observation can be explained by fact that *S. uncinata* can use rhizoids to ensure some part of water supply. Elements dissolved in soil-water may then be transported from soil to moss (Osyczka et al. 2007). Only for Cl, Ag, I, Lu, and W concentrations were greater in lichens than in mosses. The excess of I and Cl in lichens as compared to mosses can be probably an effect of organohalogen synthesis by fungal part of lichen (Matschullat et al. 1999; Gribble 2010).

**Table 3:** Results of lichens samples analysis

	Elements concentrations in lichens samples (*n* = 7*)*	
	Median (mg/kg)	Range (mg/kg)
Na	638 ± 19	435 ± 13 ÷ 8040 ± 240
Mg	794 ± 32	395 ± 16 ÷ 2060 ± 62
Al	832 ± 25	246 ± 7 ÷ 2900 ± 87
Si	12,300 ± 3700	8300 ± 2500 ÷ 24,400 ± 7300
S	5000 ± 1500	3410 ± 1000 ÷ 5940 ± 1800
Cl	1146 ± 92	426 ± 34 ÷ 15,600 ± 1200
K	2070 ± 210	1650 ± 170 ÷ 2750 ± 300
Ca	8970 ± 630	1530 ± 120 ÷ 14,900 ± 1000
Sc	0.76 ± 0.02	0.32 ± 0.01 ÷ 4.01 ± 0.12
Ti	110 ± 8	25.7 ± 5.4 ÷ 241 ± 15
V	1.95 ± 0.09	0.59 ± 0.04 ÷ 9.1 ± 0.3
Cr	2.99 ± 0.90	0.701 ± 0.210 ÷ 4.02 ± 0.68
Mn	14.2 ± 0.9	10.1 ± 0.7 ÷ 35.8 ± 2.2
Fe	441 ± 35	126 ± 14 ÷ 7140 ± 430
Ni	2.02 ± 0.61	1.43 ± 0.43 ÷ 3.48 ± 0.14
Co	0.208 ± 0.023	0.091 ± 0.013 ÷ 3.23 ± 0.26
Cu	12.7 ± 3.8	6.6 ± 2.0 ÷ 29.3 ± 8.8
Zn	14.0 ± 0.7	5.6 ± 0.3 ÷ 33.9 ± 1
Se	0.759 ± 0.053	0.428 ± 0.034 ÷ 1.05 ± 0.07
As	0.261 ± 0.010	0.21 ± 0.01 ÷ 0.578 ± 0.023
Br	41.9 ± 1.3	15.9 ± 0.5 ÷ 88.9 ± 2.7
Rb	1.04 ± 0.18	0.78 ± 0.14 ÷ 3.76 ± 0.64
Sr	30.5 ± 2.8	13.7 ± 1.4 ÷ 260 ± 23
Zr	4.61 ± 1.38	3.8 ± 1.1 ÷ 9.2 ± 2.8
Mo	0.145 ± 0.044	0.113 ± 0.034 ÷ 0.235 ± 0.071
Ag	0.79 ± 0.24	0.057 ± 0.017 ÷ 0.138 ± 0.041
Cd	< MDC	< MDC
In	0.070 ± 0.022	0.019 ± 0.007 ÷ 0.139 ± 0.046
Sb	0.0124 ± 0.0025	0.004 ± 0.002 ÷ 0.0413 ± 0.005
I	5.5 ± 2.1	1.76 ± 0.67 ÷ 7.62 ± 2.89
Ba	2.05 ± 0.33	0.78 ± 0.27 ÷ 18.2 ± 1.1
Cs	0.023 ± 0.004	0.008 ± 0.001 ÷ 0.08 ± 0.01
La	2.03 ± 0.08	0.46 ± 0.02 ÷ 4.65 ± 0.14
Ce	4.26 ± 0.29	1.12 ± 0.15 ÷ 9.84 ± 0.59
Nd	2.96 ± 0.72	0.75 ± 0.23 ÷ 6.9 ± 2.2
Sm	0.628 ± 0.031	0.127 ± 0.007 ÷ 1.67 ± 0.08
Eu	0.115 ± 0.016	0.0218 ± 0.0075 ÷ 0.448 ± 0.031
Gd	0.126 ± 0.021	0.062 ± 0.019 ÷ 0.478 ± 0.038
Tb	0.0793 ± 0.0024	0.0165 ± 0.0008 ÷ 0.231 ± 0.005
Dy	0.420 ± 0.15	0.054 ± 0.019 ÷ 1.11 ± 0.39
Tm	0.0320 ± 0.0096	0.025 ± 0.008 ÷ 0.114 ± 0.003
Yb	0.208 ± 0.023	0.063 ± 0.016 ÷ 0.669 ± 0.047
Lu	0.144 ± 0.043	0.081 ± 0.024 ÷ 0.218 ± 0.065
Hf	0.049 ± 0.015	0.015 ± 0.006 ÷ 0.197 ± 0.061
Ta	0.0057 ± 0.0017	0.0036 ± 0.0011 ÷ 0.0137 ± 0.0009
W	0.163 ± 0.049	0.105 ± 0.032 ÷ 0.275 ± 0.083
Au	0.00036 ± 0.00012	0.00012 ± 0.00005 ÷ 0.00064 ± 0.00021
Hg	0.101 ± 0.030	0.0694 ± 0.0208 ÷ 0.117 ± 0.035
Th	0.103 ± 0.006	0.0369 ± 0.0026 ÷ 0.249 ± 0.013
U	0.0392 ± 0.0035	0.0202 ± 0.0030 ÷ 0.0767 ± 0.0046

**Table 4:** Results of moss samples analysis

	Element concentrations in moss samples (*n* = 9)	
	Median (mg/kg)	Range (mg/kg)
Na	8010 ± 24	4210 ± 126 ÷ 11,300 ± 340
Mg	17,300 ± 520	6790 ± 204 ÷ 28,600 ± 570
Al	26,800 ± 610	12,400 ± 250 ÷ 50,200 ± 1000
Si	59,000 ± 14,000	16,100 ± 5600 ÷ 105,000 ± 29,000
S	27,100 ± 8100	13,700 ± 4110 ÷ 46,000 ± 14,000
Cl	512 ± 44	243 ± 24 ÷ 3700 ± 300
K	4970 ± 500	3300 ± 330 ÷ 8260 ± 740
Ca	15,900 ± 950	7540 ± 680 ÷ 23,600 ± 1200
Sc	9.45 ± 0.19	4.20 ± 0.13 ÷ 15.7 ± 0.3
Ti	1650 ± 120	765 ± 46 ÷ 2700 ± 160
V	73.2 ± 1.5	29.4 ± 0.9 ÷ 118 ± 4
Cr	40.0 ± 2.4	4.48 ± 0.99 ÷ 153 ± 5
Mn	341 ± 21	168 ± 10 ÷ 598 ± 36
Fe	22,500 ± 1100	6830 ± 410 ÷ 39,500 ± 2000
Ni	8.51 ± 1.53	3.99 ± 1.08 ÷ 65.9 ± 5.3
Co	12.4 ± 0.9	4.06 ± 0.33 ÷ 19.2 ± 1.5
Cu	53.6 ± 16.1	29.6 ± 8.9 ÷ 96.7 ± 29.1
Zn	31.2 ± 0.6	20.0 ± 0.4 ÷ 62.2 ± 1.9
Se	1.14 ± 0.08	0.515 ± 0.052 ÷ 8.43 ± 0.42
As	1.30 ± 0.09	0.363 ± 0.022 ÷ 7.77 ± 0.39
Br	67.9 ± 2.1	24.4 ± 0.7 ÷ 133 ± 4
Rb	6.52 ± 1.04	2.60 ± 0.44 ÷ 16.8 ± 2.7
Sr	167 ± 15	101 ± 9 ÷ 278 ± 25
Zr	29.7 ± 9.8	9.41 ± 3.39 ÷ 87.8 ± 27.2
Mo	0.548 ± 0.181	0.144 ± 0.061 ÷ 4.27 ± 14.09
Ag	0.133 ± 0.024	0.0552 ± 0.0049 ÷ 0.318 ± 0.032
Cd	1.04 ± 0.31	0.204 ± 0.084 ÷ 1.41 ± 0.42
In	0.121 ± 0.038	0.0981 ± 0.0304 ÷ 0.144 ± 0.045
Sb	0.0557 ± 0.0067	0.0261 ± 0.0029 ÷ 0.259 ± 0.026
I	3.42 ± 1.23	0.85 ± 0.26 ÷ 4.51 ± 1.58
Ba	74.9 ± 3.8	28.7 ± 1.7 ÷ 189 ± 10
Cs	0.363 ± 0.015	0.0906 ± 0.0073 ÷ 1.31 ± 0.04
La	5.24 ± 0.21	2.39 ± 0.09 ÷ 13.3 ± 0.4
Ce	11.6 ± 0.8	7.86 ± 0.63 ÷ 26.8 ± 1.6
Nd	6.75 ± 2.23	1.46 ± 0.43 ÷ 20.5 ± 6.8
Sm	1.44 ± 0.09	0.818 ± 0.057 ÷ 2.15 ± 0.15
Eu	0.563 ± 0.034	0.321 ± 0.032 ÷ 0.862 ± 0.051
Gd	0.510 ± 0.051	0.180 ± 0.023 ÷ 1.77 ± 0.14
Tb	0.212 ± 0.004	0.154 ± 0.005 ÷ 0.371 ± 0.007
Dy	0.937 ± 0.375	0.649 ± 0.241 ÷ 1.77 ± 0.64
Tm	0.099 ± 0.011	0.0638 ± 0.0071 ÷ 0.161 ± 0.016
Yb	0.624 ± 0.056	0.492 ± 0.049 ÷ 1.08 ± 0.09
Lu	0.089 ± 0.027	0.0274 ± 0.0082 ÷ 0.125 ± 0.038
Hf	1.06 ± 0.32	0.61 ± 0.18 ÷ 2.82 ± 0.85
Ta	0.0515 ± 0.0015	0.0404 ± 0.0024 ÷ 0.113 ± 0.003
W	0.095 ± 0.028	0.0299 ± 0.0089 ÷ 0.212 ± 0.069
Au	0.0057 ± 0.0018	0.00091 ± 0.00052 ÷ 0.0154 ± 0.0046
Hg	0.68 ± 0.20	0.56 ± 0.17 ÷ 1.06 ± 0.32
Th	0.975 ± 0.049	0.192 ± 0.012 ÷ 2.84 ± 0.14
U	0.465 ± 0.023	0.133 ± 0.008 ÷ 1.67 ± 0.08

### Rare earth elements

In this group, concentrations of 12 elements (Sc, La, Ce, Nd, Sm, Eu, Gd, Tb, Dy, Tm, Yb, Lu) were measured. Rare earth elements (REE) are naturally occurring ingredients of lithosphere commonly used for tracing geochemical processes, but also are widely applied in industry (e.g., electronics or medicine), and therefore anthropogenic contamination with REE is possible (Brioschi et al. 2013; Allajbeu et al. 2016). For lichens, the highest concentrations of REE were usually found on Red Hill and the lowest on Varueal Peak. In case of mosses, the highest concentrations were measured in samples collected near Ferraz Station and the lowest in samples from Blue Dyke. For RRE, chondrite normalization (Taylor and McLennan 1985) was applied and geochemical parameters were calculated to obtain data about their fractionation, and results are showed in Table 5.

**Table 5:** Geochemical parameters for rare earth elements (RRE) and chondrite normalized concentrations in samples and upper continental crust (UCC)

Element	Lichens normalized (mg/kg)	Mosses normalized (mg/kg)	UCC normalized (mg/kg)
La	5.45 ± 3.62	18.15 ± 10.16	84.47
Ce	4.32 ± 2.91	14.11 ± 7.53	65.83
Nd	3.95 ± 2.70	12.37 ± 7.74	37.97
Sm	2.78 ± 2.15	7.19 ± 3.51	20.35
Eu	1.79 ± 1.52	6.50 ± 2.24	11.49
Gd	0.56 ± 0.45	2.04 ± 1.61	13.07
Tb	1.54 ± 1.20	4.21 ± 1.51	12.07
Dy	1.21 ± 0.90	3.13 ± 1.37	10.24
Tm	1.28 ± 0.82	2.89 ± 0.90	8.22
Yb	1.03 ± 0.80	2.96 ± 0.96	8.06
Lu	3.83 ± 1.30	2.45 ± 0.80	8.14
Geochemical parameters*			
(Ce/Yb)n	4.53 ± 1.08	4.73 ± 1.93	8.16
(Gd/Yb)n	0.60 ± 0.19	0.64 ± 0.35	1.62
(La/Sm)n	2.17 ± 0.53	2.47 ± 0.38	4.15
δEu	1.48 ± 0.59	1.98 ± 0.61	0.70
(La/Yb)n	5.80 ± 1.92	6.05 ± 2.65	10.47
Th/Sc	0.11 ± 0.04	0.10 ± 0.07	0.75
La/Th	20.86 ± 11.97	7.82 ± 2.93	2.95
Sm/La	0.31 ± 0.07	0.26 ± 0.04	0.15
Tm/Tb	0.73 ± 0.50	0.44 ± 0.07	0.43

•See text for details

In Table 5, upper continental crust (UCC) is a reference composition (Rudnick and Gao 2004). Values of all REE concentrations are lower than reference values for UCC. For geochemical parameters, there were no significant differences between mosses and lichens, except La/Th and Tm/Tb ratios, but in these cases, standard deviation in lichens was high. That means that both groups of organisms are accumulating REE similarly. Both groups show fractionation of REE. For example, δEu is more than two times higher than reference value for UCC. Eu anomalies were calculated using equation proposed by Taylor and McLennan (1995):

1 $$ \updelta \mathrm{Eu}=\frac{{\mathrm{Eu}}_{\mathrm{n}}}{\sqrt{\left({\left[\mathrm{Sm}\right]}_{\mathrm{n}}\times {\left[\mathrm{Gd}\right]}_{\mathrm{n}}\right)}} $$

where Eu_n_, [Sm]_n_, and [Gd]_n_ are chondrite normalized concentrations of europium, samarium, and gadolinium. Similar observations were reported in other studies (Ryghaug 1983; Aubert et al. 2006) and show positive anomaly for heavy REE (Allajbeu et al. 2016). All of calculated parameters show REE distribution patterns different than UCC. The La/Th ratio higher than UCC can be evidence for sedimentary rock-originated REE’s deposited on mosses and lichens (McLennan et al. 1993).

### Heavy metals

To asses King George contamination, 15 heavy metals (V, Cr, Mn, Fe, Ni, Co, Cu, Zn, Se, As, Mo, Cd, Sb, W, and Hg) were measured. For lichen samples, most contaminated area was Marsh Airfield. Only three elements (Cd, Mo, and Se) had maximal concentrations on Red Hill. In moss samples, it was difficult to choose one most contaminated site. Highest concentrations of Mn, Fe, Co, Zn, Sb, and V were founded near Bellingshausen station. Marsh Airfield had the highest Cd concentration. Maximal concentrations of Mo, As, and Se were measured in samples collected near Machu Picchu base. Varueal Peak was a sampling site with highest Cu concentration and Lions Ramp with Cr and Ni. Median values of heavy metal concentration in lichen and moss samples from King George Island can be compared with values from Norway (Steinnes 2005; Barandovski et al. 2006), reference plant model (Markert 1991), and literature data for Antarctic region (Bargagli et al. 1999; Bargagli et al. 2000; Gonzáles et al. 2002; Smykla et al. 2005; Lim et al. 2009; Osyczka et al. 2007; Zvěřina et al. 2014; Bubach et al. 2016) (Tables 6 and 7).

**Table 6:** Concentrations of heavy metals and Se compared with literature data, reference plant (RP), and Norway moss survey (Bargagli et al. 1999; Bargagli et al. 2000; Gonzáles et al. 2002; Ganeva and Yurukova 2004; Smykla et al. 2005; Barandovski et al. 2006; Osycza et al. 2007; Lim et al. 2009; Mão de Ferro et al. 2013; Zvěřina et al. 2014; Amaro et al. 2015; Bubach et al. 2016)

Element	Moss median (mg/kg)	Literature range for moss (mg/kg)	Lichen median (mg/kg)	Literature range for lichen (mg/kg)	Norway (mg/kg)	RP (mg/kg)
V	73.2 ± 1.5	5 ÷ 75	1.95 ± 0.09	1 ÷ 75	0.92	0.50
Cr	40.0 ± 2.4	4 ÷ 9	2.99 ± 0.90	0.02 ÷ 6.8	0.55	1.50
Mn	341 ± 21	68 ÷ 390	14.2 ± 0.9	10 ÷ 180	256	200
Fe	22,500 ± 1125	3500 ÷ 21,800	441 ± 35	205 ÷ 12,670	209	150
Ni	8.51 ± 1.53	1.5 ÷ 2.8	2.02 ± 0.61	1 ÷ 5.1	1.14	1.50
Co	12.4 ± 0.9	2.6 ÷ 2.7	0.208 ± 0.023	0.02 ÷ 1.6	0.202	0.20
Cu	53.6 ± 16.1	2 ÷ 121	12.7 ± 3.8	1.8 ÷ 6.7	3.6	10
Zn	31.2 ± 0.6	6 ÷ 67	14.0 ± 0.7	0.59 ÷ 71.9	26.5	50
Se	1.14 ± 0.08	0.5 ÷ 0.6	0.759 ± 0.053	0.1 ÷ 0.3	0.093	0.02
As	1.30 ± 0.09	0.7 ÷ 38	0.261 ± 0.010	0.11 ÷ 2.3	0.33	0.1
Mo	0.548 ± 0.181	0.2 ÷ 0.7	0.145 ± 0.044	–	0.135	0.5
Cd	1.04 ± 0.31	0.05 ÷ 0.92	< MDC	0.01 ÷ 0.05	0.058	0.05
Sb	0.0557 ± 0.0067	0.03 ÷ 0.13	0.0124 ± 0.0025	–	0.033	0.1
W	0.0946 ± 0.0284	–	0.163 ± 0.049	–	0.127	0.2
Hg	0.675 ± 0.203	0.055 ÷ 0.56	0.101 ± 0.030	0.026 ÷ 0.190	0.046	0.1

**Table 7:** Factor analysis of NAA data on moss and lichen samples (Rotation method: Varimax with Kaiser normalization)

Element	Factor number			
	1	2	3	4
Na	0.597	0.670	− 0.072	0.135
Mg	0.302	0.917	− 0.008	0.021
Al	0.573	0.772	0.022	0.210
Si	0.512	0.690	0.023	0.427
S	0.442	0.833	0.057	− 0.064
Cl	− 0.125	− 0.142	− 0.107	− 0.085
K	0.736	0.280	0.139	0.359
Ca	0.093	0.791	0.434	0.134
Sc	0.497	0.828	− 0.047	0.037
Ti	0.557	0.799	− 0.030	0.129
V	0.557	0.867	− 0.032	0.174
Cr	0.423	0.604	− 0.065	− 0.346
Mn	0.093	0.898	0.010	0.190
Fe	0.306	0.840	0.005	0.146
Ni	0.459	0.640	− 0.034	− 0.322
Co	− 0.018	0.931	− 0.020	0.034
Cu	0.248	0.549	0.578	0.109
Zn	0.120	0.016	0.972	− 0.030
Se	0.037	− 0.179	0.864	0.415
As	0.527	0.306	0.183	0.744
Br	0.133	0.127	0.302	− 0.073
Rb	0.944	0.139	0.049	0.234
Sr	0.328	0.442	0.566	− 0.007
Zr	0.929	0.327	0.012	0.069
Mo	0.532	0.100	0.096	0.779
Ag	− 0.053	− 0.159	0.969	− 0.029
Cd	0.073	0.139	0.024	0.031
In	0.150	0.436	0.379	− 0.350
Sb	0.685	0.419	0.236	0.435
I	− 0.085	− 0.313	0.335	− 0.035
Ba	0.882	0.348	0.049	0.077
Cs	0.806	0.149	0.132	0.487
La	0.840	0.336	− 0.046	0.392
Ce	0.845	0.361	− 0.028	0.352
Nd	0.909	0.262	− 0.073	0.213
Sm	0.764	0.423	− 0.080	0.377
Eu	0.687	0.540	− 0.041	0.375
Gd	0.873	0.362	− 0.038	−0.171
Tb	0.556	0.639	− 0.044	0.387
Dy	0.561	0.592	0.048	0.371
Tm	0.561	0.662	− 0.032	0.216
Yb	0.591	0.667	0.074	0.172
Lu	− 0.179	− 0.354	− 0.221	− 0.092
Hf	0.905	0.391	0.000	0.033
Ta	0.822	0.489	0.122	− 0.059
W	0.073	− 0.075	− 0.283	− 0.141
Au	0.488	0.175	0.146	0.738
Hg	0.423	0.326	0.024	0.382
Th	0.887	0.217	0.379	0.068
U	0.900	0.195	0.236	0.043
Variance (%)	55.85	10.9	9.49	7.6

Obtained results are general in the range of literature data. In moss samples, however, concentrations of Cr and Ni are elevated. The same situation can be observed for Se in lichen samples. In comparison to values from Norway, concentrations of almost all heavy metals are elevated; only depositions of W and Zn are lower.

### Sources of pollutants

There are many possible sources of contamination of investigated area. The possible sources can be elevated natural background, local contamination from human activity, or long-range atmospheric transport. It was reported that As from Chilean Cu mines was found in Antarctic ice cores. Also, important source of As and Se is combustion of coal with arsenopyrite. The natural background can be also elevated due to volcanic activity (Sieprawska et al. 2015; Schwanck et al. 2016). On the other hand, Amouroux et al. (2001) described mechanism of releasing Se from oceans. In effect of biogenic activity, volatile compounds as dimethyl sulfide (DMS) and dimethyl selenide (DMSe) can be released from the ocean and later be deposited on a land surface. Oceans can be also source of Cd (Bargagli et al. 1998). For some element deposition, also local microclimate can be important and must be taken into account (Zvěřina et al. 2014). For establishing potential sources of pollution, statistical methods can be used. In our work, factor analysis was used. Based on this approach, four factors can be distinguished as an origin of pollutants in lichen and moss samples.

Factor 1 represents typical crustal composition and is associated with resuspension of soil and rock particles. Factor 2 can be probably associated with volcanic activity. Antarctic Peninsula has extensive volcanic system, and Deception Island is one of active volcanoes. Soils of King George Island have high content of tephra and ash (Deheyn et al. 2005), which probably are resuspended similar to factor 1. Factors 3 and 4 are probably connected with human activity on island and long-range transport of pollutants. There is also a possibility that some of pollutants in these factors are delivered by birds eating contaminated fishes. Pollutants could be then deposited on island with feces. That situation can be observed near Admiralty Bay where penguin colonies and ornithogenic soils are present, and arsenic concentrations were in general higher than in other sampling sites, and arsenic enriches in penguin dropping sediment (Xie and Sun 2008). Factor 4 includes Mo and As. Both of them can be local pollutants or transported to the island (Schwanck et al. 2016), where Mo may be reduced to insoluble form by bacterial activity (Ahmad et al. 2013). Chromium and Ni can be connected with combustion of diesel oil or with construction materials like stainless steel (Barałkiewicz and Siepak 1999; Kotaś and Stasicka 1999).

### SEM results

**Fig. 2:**
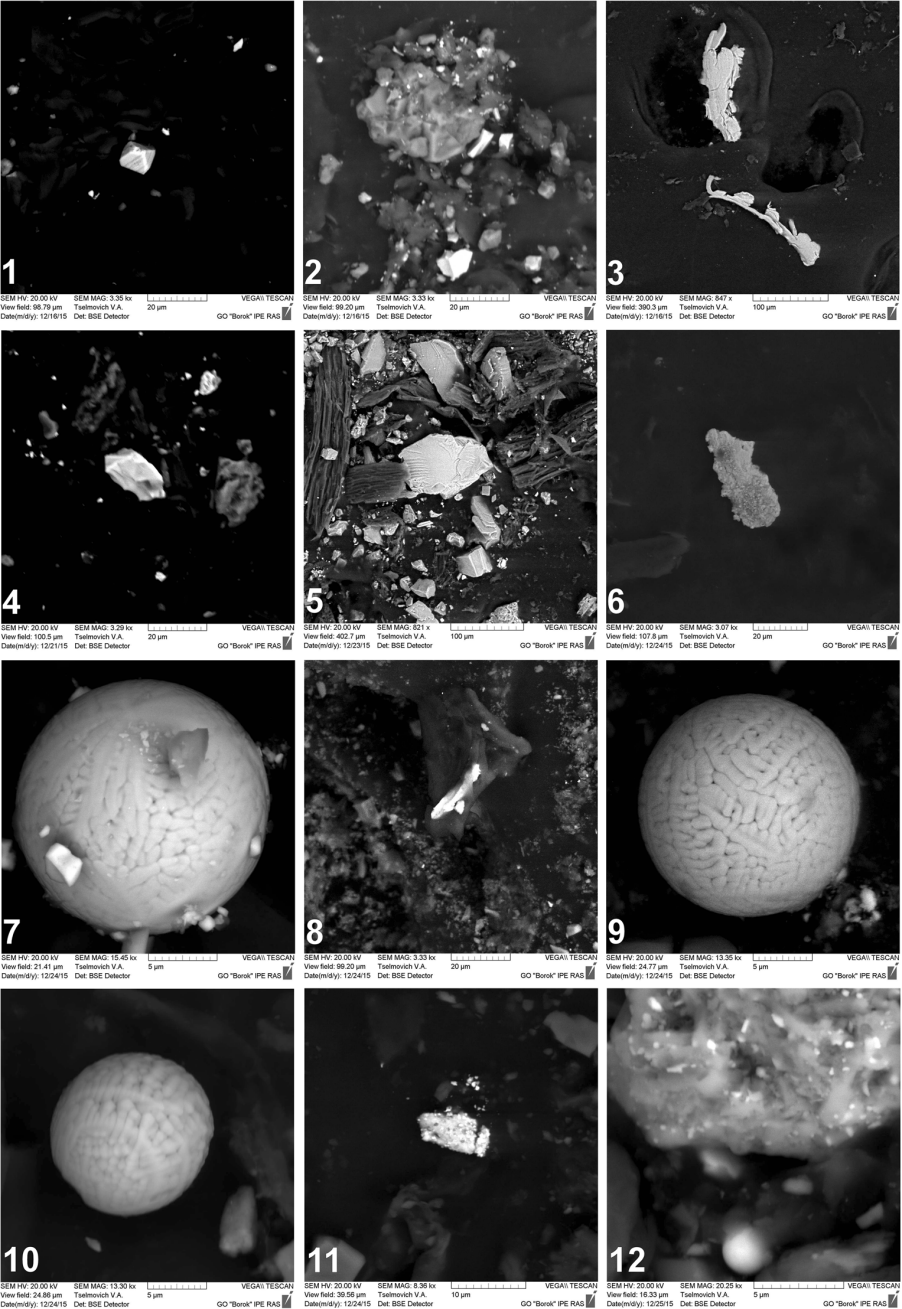
SEM photos of earth-origin (objects 1–6) and space-origin (objects 6–12) microparticles

SEM microscopy images of investigated samples of moss and lichen showed presence of volcano and cosmic-originated particles in all samples (Fig. 2). First six samples (1–6) are probably volcanic-originated. Sample 1 is titanomagnetite monocrystal. Number 2 is also titanomagnetite but with detrital structure. Number 3 is a native Al, with 5% Mg impurity. Sample number 4 is a pyrite (Fe 45%, S 48%, and O 7% by mass). Next sample (number 5) is a pyroxene and titanomagnetite. Large object in center of image is a pyroxene (44% O, 9% Mg, 2% Al, 23% Si, 13% Ca, and 10% Fe by mass), and small bright spots are titanomagnetites (35% O, 2% Al, 1% Si, 6% Ti, and 56% Fe); sample 6 is native iron. Samples 7, 9, 10, and 12 are spherical object space origin classified due to their dimensions as IDP and with chemical composition of Fe and O, without Ti. Samples 8 and 11 have differed chemical composition. In sample 8, we have 6% O, 16% Cr, and 78% of Fe and its chemical composition can be evidence for cosmic origin (Korchagin et al. 2010). Finally, sample 11 is made from Ti (2%), Ni (4%), Cu (49%), Zn (17%), and W (28%) and is a rare example of cosmic particle with Cu-Zn alloy (Korchagin et al. 2010) where Ti can be contaminated from basalts (Pechersky et al. 2015a, b). The spherical IDPs found in our samples are type I microspherules (Brownlee et al. 1997) and are composed mainly from Fe_3_O_4_ and FeO (Engrand et al. 2005). As the average flux of cosmic dust is 1/m^2^/d for 10 μm particles and 1/m^2^/y for 100 μm (McDonnell 1978; McDonnell et al. 1984), total annual deposition of cosmic particles on King George Island (1150 km^2^) can be estimated as 4.2∙10^11^ 10 μm particles and 1.15∙10^9^ 100 μm particles.

## Summary

In this study, 50 element concentrations were determined using instrumental neutron activation analysis in moss and lichen samples. Based on INAA and statistical results (PCA analysis), a few possible sources of pollution of King George Island were suggested. Also, presence of extraterrestrial material in investigated samples was confirmed by SEM microscopy analysis.

## Electronic supplementary material

ESM 1 (DOCX 34 kb).
