# Supplementary material for: Determination of element composition and extraterrestrial material occurrence in moss and lichen samples from King George Island (Antarctica) using reactor neutron activation analysis and SEM microscopy
Source: Environ Sci Pollut Res Int. 2017 Oct 18;25(1):436–46. doi: 10.1007/s11356-017-0431-2 (PMC5756565; doi:10.1007/s11356-017-0431-2)
Supplement: Supplementary file 1 — (DOCX 34 kb). [file 11356_2017_431_MOESM1_ESM.docx]

Supplementary data – elements concentrations by species and sites

| Element | Element concentration [mg kg^-1^] | | | | | |
| --- | --- | --- | --- | --- | --- | --- |
|  | Marsch Airfield | | Ferraz Station | | Bellingshausen Station | |
|  | *Usnea antarctica* | *Sanionia uncinata* | *Usnea antarctica* | *Sanionia uncinata* | *Usnea aurantiaco-atra* | *Sanionia uncinata* |
| Na | 4100 ± 123 | 7440 ± 223 | 435 ± 13 | 11300 ± 339 | 2150 ± 65 | 8870 ± 266 |
| Mg | 2060 ± 58 | 15700 ± 390 | 559 ± 22 | 17300 ± 434 | 666 ± 26 | 28600 ± 695 |
| Al | 2900 ± 75 | 26700 ± 617 | 616 ± 16 | 47000 ± 1095 | 450 ± 12 | 50200 ± 1165 |
| Si | 24400 ± 7320 | 34300 ± 10626 | 9920 ± 2976 | 85500 ± 22631 | 12800 ± 3840 | 99300 ± 22878 |
| S | 4820 ± 1446 | 30100 ± 9030 | 4990 ± 1497 | 41100 ± 12330 | 5830 ± 1749 | 45900 ± 13770 |
| Cl | 6530 ± 536 | 3740 ± 305 | 487 ± 41 | 512 ± 44 | 3970 ± 326 | 243 ± 23 |
| K | 2130 ± 221 | 4970 ± 515 | 2050 ± 190 | 8200 ± 768 | 2360 ± 208 | 3300 ± 332 |
| Ca | 14900 ± 1013 | 14900 ± 831 | 4020 ± 292 | 12700 ± 735 | 8150 ± 562 | 23600 ± 1267 |
| Sc | 4.01 ± 0.11 | 12.1 ± 0.2 | 0.61 ± 0.02 | 13 ± 1 | 1.4 ± 0.03 | 15.7 ± 0.3 |
| Ti | 220 ± 17 | 1390 ± 100 | 74.9 ± 4.6 | 2580 ± 163 | 45.4 ± 5.8 | 2700 ± 175 |
| V | 9.1 ± 0.3 | 76.3 ± 2.5 | 1.3 ± 0.6 | 85.7 ± 2.9 | 1.37 ± 0.07 | 118 ± 4 |
| Cr | 4.02 ± 0.69 | 11.6 ± 2.1 | 2.94 ± 0.88 | 17.1 ± 0.8 | 3.04 ± 0.91 | 40 ± 2 |
| Mn | 36 ± 2 | 382 ± 23 | 8.73 ± 0.59 | 317 ± 19 | 10.4 ± 0.8 | 598 ± 36 |
| Fe | 7140 ± 394 | 24700 ± 1336 | 404 ± 34 | 26700 ± 1441 | 365 ± 29 | 39500 ± 2129 |
| Ni | 3.48 ± 1.04 | 4.09 ± 0.82 | 1.95 ± 0.59 | 5.62 ± 0.95 | 2.11 ± 0.63 | 20.6 ± 1.9 |
| Co | 3.23 ± 0.26 | 12.9 ± 1.1 | 0.133 ± 0.016 | 9.23 ± 0.76 | 0.185 ± 0.019 | 19.2 ± 1.6 |
| Cu | 29.3 ± 8.8 | 48.4 ± 14.5 | 6.62 ± 1.99 | 66.5 ± 19.9 | 15.6 ± 4.7 | 96.7 ± 29.1 |
| Zn | 22.9 ± 0.8 | 34 ± 1 | 14.9 ± 0.68 | 38.7 ± 1.3 | 20.7 ± 0.7 | 62.2 ± 1.7 |
| Se | 0.827 ± 0.055 | 0.764 ± 0.067 | 0.991 ± 0.059 | 1.63 ± 0.09 | 0.569 ± 0.034 | 1.19 ± 0.08 |
| As | 0.543 ± 0.017 | 1.3 ± 0.1 | 0.223 ± 0.009 | 3.96 ± 0.21 | 0.215 ± 0.009 | 4.69 ± 0.24 |
| Br | 51.5 ± 1.4 | 105 ± 3 | 15.9 ± 0.4 | 67.9 ± 1.7 | 20.8 ± 0.6 | 60.8 ± 1.6 |
| Rb | 1.57 ± 0.27 | 4.37 ± 0.72 | 1.32 ± 0.23 | 25.8 ± 4.2 | 1.12 ± 0.19 | 5.67 ± 0.95 |
| Sr | 260 ± 22 | 278 ± 25 | 37.6 ± 3.4 | 207 ± 18 | 23.4 ± 2.34 | 185 ± 17 |
| Zr | 9.22 ± 2.77 | 16.6 ± 5.4 | 4.01 ± 1.21 | 87.8 ± 26.8 | 6.1 ± 1.8 | 32.7 ± 10.1 |
| Mo | 0.195 ± 0.059 | 0.233 ± 0.128 | 0.113 ± 0.034 | 1.66 ± 0.55 | 0.119 ± 0.036 | 0.999 ± 0.329 |
| Ag | 0.138 ± 0.041 | 0.0877 ± 0.0263 | 0.0 776 ± 0.0233 | 0.133 ± 0.023 | 0.0921 ± 0.0276 | 0.318 ± 0.033 |
| Cd | <MDC | <MDC | <MDC | 1.03 ± 0.28 | <MDC | <MDC |
| In | 0.103 ± 0.032 | 0.121 ± 0.038 | 0.0369 ± 0.0123 | 0.132 ± 0.041 | 0.102 ± 0.031 | 0.13 ± 0.04 |
| Sb | 0.0349 ± 0.0048 | 0.0373 ± 0.0049 | 0.0109 ± 0.0023 | 0.256 ± 0.025 | 0.0213 ± 0.0034 | 0.259 ± 0.026 |
| I | 7.62 ± 2.89 | 4.51 ± 1.59 | 2.11 ± 0.81 | 3.66 ± 1.29 | 2.75 ± 1.05 | 3.42 ± 1.22 |
| Ba | 18.2 ± 1.04 | 68.1 ± 3.6 | 2.31 ± 0.35 | 189 ± 9 | 1.07 ± 0.65 | 74.9 ± 3.6 |
| Cs | 0.074 ± 0.005 | 0.243 ± 0.011 | 0.0244 ± 0.0029 | 1.2 ± 0.1 | 0.0215 ± 0.0031 | 0.26 ± 0.02 |
| La | 2.17 ± 0.08 | 4.95 ± 0.19 | 2.51 ± 0.08 | 13.3 ± 0.5 | 1.89 ± 0.07 | 6.97 ± 0.25 |
| Ce | 5.25 ± 0.44 | 10.1 ± 0.74 | 4.34 ± 0.28 | 26.8 ± 1.6 | 4.17 ± 0.27 | 14.1 ± 0.9 |
| Nd | 3.18 ± 1.11 | 7.56 ± 2.56 | 3.16 ± 1.06 | 20.5 ± 6.8 | 2.98 ± 1.04 | 6.63 ± 2.12 |
| Sm | 0.762 ± 0.036 | 1.44 ± 0.09 | 0.767 ± 0.036 | 3.1 ± 0.2 | 0.81 ± 0.04 | 2.15 ± 0.15 |
| Eu | 0.201 ± 0.027 | 0.622 ± 0.032 | 0.0968 ± 0.0205 | 0.862 ± 0.053 | 0.185 ± 0.017 | 0.698 ± 0.045 |
| Gd | 0.205 ± 0.029 | 0.51 ± 0.05 | 0.208 ± 0.024 | 1.77 ± 0.14 | 0.153 ± 0.022 | 0.854 ± 0.076 |
| Tb | 0.113 ± 0.003 | 0.289 ±0.007 | 0.0891 ± 0.0025 | 0.324 ± 0.007 | 0.126 ± 0.003 | 0.371 ± 0.009 |
| Dy | 0.487 ± 0.177 | 1.55 ± 0.56 | 0.292 ± 0.105 | 1.93 ± 0.68 | 0.747 ± 0.259 | 1.77 ± 0.63 |
| Tm | 0.0621 ± 0.0167 | 0.121 ± 0.015 | 0.0306 ± 0.0103 | 0.146 ± 0.015 | 0.0475 ± 0.0125 | 0.161 ± 0.016 |
| Yb | 0.3 ± 0.04 | 0.914 ± 0.073 | 0.207 ± 0.022 | 1.11 ± 0.08 | 0.384 ± 0.032 | 1.08 ± 0.08 |
| Lu | 0.152 ± 0.046 | 0.123 ± 0.037 | 0.0811 ± 0.0243 | 0.0901 ± 0.027 | 0.124 ± 0.037 | 0.0875 ± 0.0263 |
| Hf | 0.197 ± 0.061 | 0.761 ± 0.231 | 0.037 ±0.012 | 2.82 ± 0.85 | 0.0619 ± 0.0199 | 1.09 ± 0.33 |
| Ta | 0.00661 ± 0.00088 | 0.0404 ± 0.0023 | 0.00572 ± 0.00172 | 0.113 ± 0.003 | 0.00357 ± 0.00107 | 0.057 ± 0.002 |
| W | 0.186 ± 0.056 | 0.0946 ± 0.0283 | 0.105 ± 0.032 | 0.201 ± 0.062 | 0.124 ± 0.037 | 0.212 ± 0.069 |
| Au | 0.00064 ± 0.00020 | 0.00597 ± 0.00196 | 0.00012 ± 0.00004 | 0.00566 ± 0.00176 | 0.00017 ± 0.00006 | 0.00301 ± 0.0009 |
| Hg | 0.117 ± 0.035 | 0.768 ± 0.231 | 0.0968 ± 0.0291 | 0.814 ± 0.244 | 0.232 ± 0.069 | 0.612 ± 0.184 |
| Th | 0.249 ± 0.013 | 0.6 ± 0.1 | 0.096 ± 0.006 | 2.84 ± 0.15 | 0.11 ± 0.01 | 0.975 ± 0.051 |
| U | 0,0767 ± 0.0048 | 0.304 ± 0.017 | 0.0513 ± 0.0031 | 1.67 ± 0.08 | 0.0365 ± 0.0032 | 0.465 ± 0.025 |

| Element | Element concentration [mg kg^-1^] | | | | | |
| --- | --- | --- | --- | --- | --- | --- |
|  | Hennequin Point | | Penguin Island | | Vaureal Peak | |
|  | *Usnea aurantiaco-atra* | *Sanionia uncinata* | *Usnea antarctica* | *Sanionia uncinata* | *Usnea antarctica* | *Sanionia uncinata* |
| Na | 546 ± 17 | 5860 ± 176 | 569 ± 17 | 7200 ± 216 | 580 ± 17 | 8010 ± 240 |
| Mg | 319 ± 14 | 7340 ± 195 | 813 ± 29 | 20000 ± 484 | 775 ± 28 | 22300 ± 542 |
| Al | 246 ± 7 | 15200 ± 351 | 1370 ± 35 | 20300 ± 473 | 664 ± 17 | 28800 ± 674 |
| Si | 8340 ± 2502 | 27500 ± 6707 | 14400 ± 4320 | 59300 ± 14036 | 11300 ± 3390 | 68600 ± 16430 |
| S | 4910 ± 1473 | 19100 ± 5730 | 4990 ± 1497 | 27100 ± 8130 | 5940 ± 1782 | 39100 ± 11730 |
| Cl | 832 ± 70 | 1230 ± 102 | 426 ± 35 | 1040 ± 86 | 981 ± 82 | 470 ± 41 |
| K | 1650 ± 161 | 5510 ± 501 | 1770 ± 213 | 4800 ± 463 | 2750 ± 308 | 5870 ± 651 |
| Ca | 12300 ± 839 | 7540 ± 451 | 10200 ± 699 | 16100 ± 894 | 1530 ± 128 | 17100 ± 956 |
| Sc | 0.333 ± 0.011 | 8.94 ± 0.18 | 0.45 ± 0.01 | 8.84 ± 0.18 | 0.318 ± 0.009 | 9.45 ± 0.19 |
| Ti | 25.7 ± 5.4 | 918 ± 68 | 146 ± 11 | 1680 ± 109 | 47.6 ± 9.7 | 1530 ± 116 |
| V | 0.587 ± 0.041 | 35 ± 1 | 4.48 ± 0.16 | 69.5 ± 2.3 | 1.43 ± 0.05 | 73.2 ± 2.4 |
| Cr | 2.57 ± 0.77 | 47.4 ± 2.4 | 0.701 ± 0.211 | 122 ± 4 | 0.731 ± 0.219 | 80.4 ± 2.9 |
| Mn | 10.1 ± 0.7 | 168 ± 10 | 19.4 ± 1.2 | 301 ± 18 | 10.4 ± 0.712 | 402 ± 24 |
| Fe | 126 ± 15 | 18700 ± 1011 | 452 ± 32 | 20900 ± 1129 | 431 ± 31 | 19700 ± 1070 |
| Ni | 1.45 ± 0.44 | 4.77 ± 1.57 | 1.65 ± 0.49 | 60.7 ± 5.1 | 1.42 ± 0.43 | 33.7 ± 3.1 |
| Co | 0.0911 ± 0.0124 | 6.2 ± 0.5 | 0.141 ± 0.016 | 13.6 ± 1.1 | 0.23 ± 0.03 | 12.4 ± 1.1 |
| Cu | 10.2 ± 3.1 | 29.6 ± 8.9 | 15.2 ± 4.6 | 53.6 ± 16.1 | 9.12 ± 2.74 | 146 ± 44 |
| Zn | 3.39 ± 0.27 | 29.2 ± 1.1 | 4.58 ± 0.33 | 26.8 ± 0.9 | 5.61 ± 0.33 | 31.2 ± 0.7 |
| Se | 0.498 ± 0.035 | 1.14 ± 0.07 | 1.03 ± 0.06 | 0.555 ± 0.085 | 0.69 ± 0.05 | 0.832 ± 0.065 |
| As | 0.241 ± 0.009 | 1.72 ± 0.11 | 0.21 ± 0.01 | 0.363 ± 0.021 | 0.578 ± 0.022 | 1.07 ± 0.07 |
| Br | 19.3 ± 0.5 | 122 ± 3 | 52.6 ± 1.4 | 24.4 ± 0.6 | 88.9 ± 2.4 | 133 ± 4 |
| Rb | 0.842 ± 0.147 | 11.2 ± 1.9 | 0.799 ± 0.141 | 3.02 ± 0.52 | 0.954 ± 0.165 | 6.52 ± 1.07 |
| Sr | 28.9 ± 2.7 | 186 ± 16 | 24.9 ± 2.4 | 167 ± 15 | 13.7 ± 1.4 | 158 ± 14 |
| Zr | 4.06 ± 1.22 | 41.9 ± 12.7 | 4.71 ± 1.41 | 17.7 ± 5.9 | 3.8 ± 1.1 | 29.6 ± 9.3 |
| Mo | 0.116 ± 0.035 | 0.664 ± 0.234 | 0.178 ± 0.053 | 0.358 ± 0.118 | 0.171 ± 0.051 | 0.231 ± 0.102 |
| Ag | 0.0596 ± 0.0179 | 0.0724 ± 0.0185 | 0.0682 ± 0.025 | 0.0869 ± 0.0261 | 0.0574 ± 0.0172 | 0.196 ± 0.059 |
| Cd | <MDC | <MDC | <MDC | <MDC | <MDC | <MDC |
| In | 0.0216 ± 0.0069 | 0.0981 ± 0.0302 | 0.149 ± 0.045 | 0.132 ± 0.041 | 0.0631 ± 0.0193 | 0.144 ± 0.045 |
| Sb | 0.00447 ± 0.00159 | 0.107 ± 0.011 | 0.0118 ± 0.0021 | 0.0261 ± 0.0029 | 0.0102 ± 0.0023 | 0.0557 ± 0.0066 |
| I | 1.76 ± 0.67 | 4.36 ± 1.54 | 5.37 ± 2.03 | 0.85 ± 0.29 | 6.33 ± 2.41 | 4.24 ± 1.05 |
| Ba | 0.781 ± 0.274 | 132 ± 6 | 1.79 ± 0.31 | 46 ± 2 | 1.31 ± 0.27 | 65.2 ± 3.2 |
| Cs | 0.008 ± 0.001 | 0.377 ± 0.016 | 0.0137 ± 0.0025 | 0.0906 ± 0.0068 | 0.0162 ± 0.0021 | 0.363 ± 0.016 |
| La | 1.28 ± 0.05 | 6.83 ± 0.24 | 2.43 ± 0.08 | 3.35 ± 0.12 | 0.463 ± 0.024 | 4.82 ± 0.19 |
| Ce | 2.61 ± 0.19 | 13.5 ± 0.9 | 4.59 ± 0.28 | 7.86 ± 0.61 | 1.18 ± 0.16 | 9.52 ± 0.71 |
| Nd | 1.54 ± 0.57 | 10 ± 3 | 2.94 ± 1.13 | 6.08 ± 2.03 | 0.749 ±0.225 | 6.75 ± 2.26 |
| Sm | 0.373 ± 0.018 | 1.45 ± 0.09 | 0.493 ± 0.023 | 0.856 ± 0.057 | 0.127 ± 0.006 | 1.11 ± 0.07 |
| Eu | 0.0864 ± 0.0174 | 0.536 ± 0.032 | 0.134 ± 0.011 | 0.321 ± 0.032 | 0.0726 ± 0.0218 | 0.459 ± 0.026 |
| Gd | 0.098 ± 0.019 | 0.823 ± 0.075 | 0.099 ± 0.019 | 0.264 ± 0.025 | 0.0616 ± 0.0185 | 0.376 ± 0.045 |
| Tb | 0.0477 ± 0.0016 | 0.212 ± 0.005 | 0.0695 ± 0.0021 | 0.154 ± 0.004 | 0.0165 ± 0.0008 | 0.191 ± 0.0.005 |
| Dy | 0.344 ± 0.123 | 0.551 ± 0.237 | 0.522 ± 0.185 | 0.649 ± 0.239 | 0.054 ± 0.019 | 0.844 ± 0.326 |
| Tm | 0.0318 ± 0.00954 | 0.0942 ± 0.0102 | 0.0325 ± 0.0102 | 0.0638 ± 0.0072 | 0.0295 ± 0.0088 | 0.0782 ± 0.0114 |
| Yb | 0.118 ± 0.017 | 0.624 ± 0.053 | 0.208 ± 0.021 | 0.492 ± 0.051 | 0.0867 ± 0.0261 | 0.593 ± 0.049 |
| Lu | 0.087 ± 0.026 | 0.125 ± 0.038 | 0.164 ± 0.049 | 0.0274 ± 0.0082 | 0.205 ± 0.062 | 0.125 ± 0.038 |
| Hf | 0.0246 ± 0.0083 | 1.36 ± 0.41 | 0.015 ± 0.006 | 0.608 ± 0.184 | 0.0336 ± 0.0101 | 0.907 ± 0.273 |
| Ta | 0.0052 ± 0.0016 | 0.0649 ± 0.0027 | 0.00563 ± 0.00169 | 0.0515 ± 0.0018 | 0.00522 ± 0.00157 | 0.0506 ± 0.0025 |
| W | 0.112 ± 0.034 | 0.116 ± 0.035 | 0.213 ± 0.064 | 0.0299 ± 0.0089 | 0.275 ± 0.083 | 0.122 ± 0.036 |
| Au | 0.00038 ± 0.00012 | 0.00585 ± 0.00176 | 0.00032 ± 0.00011 | 0.00141 ± 0.00047 | 0.00053 ± 0.00016 | 0.00589 ± 0.00171 |
| Hg | 0.215 ± 0.065 | 0.675 ± 0.203 | 0.0749 ± 0.0225 | 0.556 ± 0.167 | 0.0694 ± 0.0208 | 0.646 ± 0.194 |
| Th | 0.0342 ± 0.0026 | 1.28 ± 0.07 | 0.0751 ± 0.0046 | 0.278 ± 0.015 | 0.0396 ± 0.0028 | 0.648 ± 0.036 |
| U | 0.0264 ± 0.0029 | 0.582 ± 0.029 | 0.0202 ± 0.0029 | 0.168 ± 0.009 | 0.0418 ± 0.0036 | 0.361 ± |

| Element | Element concentration [mg kg^-1^] | | | |
| --- | --- | --- | --- | --- |
|  | Red Hill | Machu Picchu | Blue Dyke | Lions Rump |
|  | *Usnea antarctica* | *Sanionia uncinata* | *Sanionia uncinata* | *Sanionia uncinata* |
| Na | 8040 ± 241 | 8590 ± 258 | 4210 ± 126 | 8300 ± 249 |
| Mg | 1480 ± 49 | 12500 ± 320 | 6790 ± 178 | 24200 ± 576 |
| Al. | 1000 ± 26 | 31500 ± 725 | 12400 ± 284 | 26800 ± 611 |
| Si | 20200 ± 6060 | 105000 ± 29190 | 16100 ± 5635 | 44500 ± 15575 |
| S | 3410 ± 1023 | 13700 ± 4110 | 14000 ± 4200 | 25200 ± 7560 |
| Cl | 15600 ± 1279 | 456 ± 40 | 1170 ± 97 | 477 ± 42 |
| K | 1750 ± 216 | 8260 ± 775 | 3910 ± 381 | 4090 ± 381 |
| Ca | 9790 ± 675 | 15900 ± 879 | 8870 ± 507 | 18800 ± 1004 |
| Sc | 1.96 ± 0.04 | 7.56 ± 0.15 | 4.2 ± 0.1 | 11.7 ± 0.2 |
| Ti | 161 ± 19 | 1650 ± 108 | 765 ± 48 | 1790 ± 108 |
| V | 2.46 ± 0.11 | 71.2 ± 2.2 | 29.4 ± 0.9 | 78.8 ± 2.5 |
| Cr | 3.56 ± 1.07 | 9.51 ± 1.06 | 4.48 ± 0.97 | 153 ± 5 |
| Mn | 18 ± 1 | 341 ± 20 | 176 ± 11 | 441 ± 26 |
| Fe | 571 ± 41 | 22500 ± 1215 | 6830 ± 376 | 26700 ± 1442 |
| Ni | 2.48 ± 0.75 | 3.99 ± 1.07 | 8.51 ± 1.53 | 65.9 ± 5.6 |
| Co | 0.246 ± 0.024 | 8.01 ± 0.66 | 4.06 ± 0.34 | 15.4 ± 1.3 |
| Cu | 33.1 ± 9.9 | 51.2 ± 15.4 | 67.8 ± 20.34 | 25.5 ± 7.7 |
| Zn | 13.1 ± 0.6 | 25.6 ± 0.83 | 20.1 ± 0.4 | 33.6 ± 1.2 |
| Se | 1.05 ± 0.07 | 8.43 ± 0.39 | 1.4 ± 0.1 | 0.515 ± 0.051 |
| As | 0.536 ± 0.0194 | 7.77 ± 0.39 | 0.871 ± 0.047 | 0.784 ± 0.051 |
| Br | 81.1 ± 2.2 | 35.8 ± 0.9 | 84.7 ± 2.2 | 35.6 ± 0.9 |
| Rb | 0.915 ± 0.161 | 16.8 ± 2.8 | 2.61 ± 0.44 | 7.63 ± 1.26 |
| Sr | 44.6 ± 4.1 | 144 ± 13 | 101 ± 10 | 154 ± 14 |
| Zr | 4.86 ± 1.46 | 41 ± 13 | 9.4 ± 3.4 | 29.7 ± 9.9 |
| Mo | 0.235 ± 0.071 | 4.27 ± 1.39 | 0.144 ± 0.0608 | 0.548 ± 0.181 |
| Ag | 0.106 ± 0.032 | 0.164 ± 0.026 | 0.213 ± 0.024 | 0.0552 ±0.0052 |
| Cd | <MDC | 0.383 ± 0.188 | 1.04 ± 0.31 | 1.41 ± 0.42 |
| In | 0.077 ± 0.024 | 0.0255 ± 0.0097 | 0.0314 ± 0.0112 | 0.0456 ± 0.0137 |
| Sb | 0.0412 ± 0.0053 | 0.237 ± 0.023 | 0.0296 ± 0.0041 | 0.0548 ± 0.0064 |
| I | 7.71 ± 2.94 | 3.4 ± 1.2 | 2.58 ± 0.92 | 1.71 ± 0.65 |
| Ba | 3.46 ± 0.49 | 104 ± 5 | 28.7 ± 1.7 | 95.4 ± 4.5 |
| Cs | 0.0235 ± 0.0037 | 1.31 ± 0.04 | 0.214 ± 0.009 | 0.474 ± 0.017 |
| La | 4.65 ± 0.16 | 12.1 ± 0.4 | 2.39 ± 0.09 | 5.24 ± 0.19 |
| Ce | 9.84 ± 0.55 | 23.4 ± 1.4 | 4.72 ± 0.31 | 11.6 ± 0.8 |
| Nd | 6.86 ± 2.21 | 13.9 ± 4.4 | 1.46 ± 0.44 | 6.3 ± 2.1 |
| Sm | 1.67 ± 0.08 | 2.7 ± 0.18 | 0.818 ± 0.056 | 1.32 ± 0.09 |
| Eu | 0.448 ± 0.031 | 0.802 ± 0.046 | 0.358 ± 0.028 | 0.404 ± 0.027 |
| Gd | 0.478 ± 0.038 | 0.273 ± 0.026 | 0.181 ± 0.023 | 0.575 ± 0.055 |
| Tb | 0.231 ± 0.005 | 0.341 ± 0.008 | 0.129 ± 0.004 | 0.191 ± 0.005 |
| Dy | 1.11 ± 0.39 | 1.61 ± 0.58 | 0.889 ± 0.329 | 0.937 ± 0.371 |
| Tm | 0.114 ± 0.025 | 0.111 ± 0.012 | 0.075 ± 0.008 | 0.0989 ± 0.0105 |
| Yb | 0.669 ± 0.049 | 0.703 ± 0.056 | 0.499 ± 0.043 | 0.611 ± 0.049 |
| Lu | 0.218 ± 0.065 | 0.0864 ± 0.0259 | 0.0858 ± 0.0257 | 0.0887 ± 0.0266 |
| Hf | 0.651 ± 0.021 | 1.17 ± 0.35 | 0.325 ± 0.099 | 1.06 ± 0.32 |
| Ta | 0.00638 ± 0.00191 | 0.0474 ± 0.0018 | 0.00208 ± 0.00015 | 0.0552 ± 0.002 |
| W | 0.228 ± 0.068 | 0.0721 ± 0.0 | 0.0509 ± 0.0153 | 0.0606 ± 0.0182 |
| Au | 0.00039 ± 0.00012 | 0.0154 ± 0.0047 | 0.00162 ± 0.00064 | 0.00091 ± 0.00034 |
| Hg | 0.0958 ± 0.0287 | 1.06 ± 0.32 | 0.871 ± 0.261 | 0.571 ± 0.171 |
| Th | 0.162 ± 0.009 | 1.36 ± 0.07 | 0.192 ± 0.011 | 1.17 ± 0.06 |
| U | 0.111 ± 0.007 | 0.663 ± 0.034 | 0.133 ± 0.008 | 0.515 ± 0.027 |
